# Mapping the Diagnosis Axis of an Interface Terminology to the NANDA International Taxonomy

**DOI:** 10.5402/2012/676905

**Published:** 2012-07-04

**Authors:** Maria-Eulàlia Juvé Udina, Maribel Gonzalez Samartino, Cristina Matud Calvo

**Affiliations:** ^1^School of Nursing, University of Barcelona, Campus of Bellvitge, Feixa Llarga s/n, 08907 Hospitalet de Llobregat, Spain; ^2^Nursing Information Systems Department, University Hospital of Bellvitge, 08907 Hospitalet de Llobregat, Spain

## Abstract

*Background*. Nursing terminologies are designed to support nursing practice but, as with any other clinical tool, they should be evaluated. Cross-mapping is a formal method for examining the validity of the existing controlled vocabularies. *Objectives*. The study aims to assess the inclusiveness and expressiveness of the nursing diagnosis axis of a newly implemented interface terminology by cross-mapping with the NANDA-I taxonomy. *Design/Methods*. The study applied a descriptive design, using a cross-sectional, bidirectional mapping strategy. The sample included 728 concepts from both vocabularies. Concept cross-mapping was carried out to identify one-to-one, negative, and hierarchical connections. The analysis was conducted using descriptive statistics. *Results*. Agreement of the raters' mapping achieved 97%. More than 60% of the nursing diagnosis concepts in the NANDA-I taxonomy were mapped to concepts in the diagnosis axis of the new interface terminology; 71.1% were reversely mapped. *Conclusions*. Main results for outcome measures suggest that the diagnosis axis of this interface terminology meets the validity criterion of cross-mapping when mapped from and to the NANDA-I taxonomy.

## 1. Introduction

Language plays an important role in defining what nurses do and why they do it. In recent decades, language systems have become a priority for international nursing agendas. Standardized controlled vocabularies are a means to develop, express, and understand nursing phenomena and actions through concepts; to quote Matney et al. “*structured nursing terminologies are needed to drive, document and evaluate nursing practice*” [1]. 

The use of electronic health records and information systems at all levels of the healthcare agencies is now widespread all over the world. In order to optimize the efficiency of these records and systems and to facilitate the exchange of information among professionals and institutions, they must be based on controlled vocabularies [2, 3]; as Müller-Staub et al. explain “*standardized computer-compatible professional terminology is becoming a requirement, especially by institutions and healthcare systems that bear the costs of health care*” [4]. 

Controlled nursing vocabularies can be implemented as interface terminologies at the point of care and as administrative terminologies to retrieve nursing clinical data in order to support decision-making [4–6]. To date, twelve nursing terminologies and data sets have been recognized by the American Nurses Association (ANA) for supporting nursing practice: the North American Nursing Diagnosis Association International Taxonomy (NANDA-I), the Nursing Interventions Classification (NIC), the Clinical Care Classification (CCC), the Omaha System, the Nursing Outcomes Classification (NOC), the Nursing Management Minimum Data Set (NMMDS), the Perioperative Nursing Data Set (PNDS), SNOMED Clinical Terms, the Nursing Minimum Data Set (NMDS), the International Classification for Nursing Practice (ICNP) from the International Council of Nurses, the ABCcodes, and the Logical Observation Identifiers Names and Codes (LOINC) [7]. 

The North American Nursing Diagnosis Association International Taxonomy (NANDA-I) is considered the most widely used and best researched nursing diagnosis vocabulary [8]. However, its use is not universal and previous studies have reported a number of issues that have affected its implementation in clinical practice. First, to document patients' problems and responses, nurses may use terms other than those in the NANDA-I Taxonomy; terms in this taxonomy labeling the nursing diagnoses are often complex, too abstract, or insufficiently specific to accurately reflect nurses' judgments of patient status [2, 9]. Second, developers who pioneered the taxonomy construction did not aim to design a controlled computer-compatible vocabulary but to demonstrate the autonomy of the nursing profession and to differentiate it from medicine and other healthcare disciplines. For years, this has been the main *raison d'être* of this terminology. Among the other aims of its development and advancement were to encourage critical thinking among nurses and to introduce systematic methods in order to reflect an individualistic nursing approach to patient care. However, almost 40 years later, both the application of these methods and the use of the NANDA-I nursing diagnoses in clinical practice are still deficient and their usefulness has been debated at length within the international nursing community [9, 10]. 

Despite these issues, nursing diagnosis concepts are needed to guide nursing practice, to increase the consistency of nursing care descriptions, to highlight the influence of nursing services on patients' outcomes, and to explain why nurses do what they do, for and with patients and families [10, 11]. 

The emphasis on the importance of establishing electronic health record systems for any care site has powered the emergence of clinical interface terminologies [12, 13]. 

An interface terminology, also named an application, entry, or colloquial terminology, aims to facilitate the interaction between the terminological system and the end-users of the electronic health records by using a close-to-natural language. 

Trent Rosenbloom et al. defined an interface terminology as a “*systematic collection of health care-related terms that supports clinicians' entry of patient-related information into computer programs” and state that the “electronic health record systems depend on interface terminologies for successful implementation in clinical settings because such terminologies provide the translation from clinicians' own natural language expressions into the more structured representations required by application programs*” [13].

This paper focuses on a nursing interface multiaxial terminology for representing nursing phenomena, implemented in the electronic health records at 11 hospitals in Catalonia (Spain). Termed ATIC, the Catalan acronym for Architecture, Terminology, Interface-Information-Nursing (*Infermeria*) and Knowledge (*Coneixement*), the evolving status of its coverage and structure and its philosophical and theoretical background have been described elsewhere [14–16].

Like any other clinical tool, nursing terminologies should be evaluated for their validity, reliability, and applicability to the practice setting. 

Mapping one terminology to another or to others is a validity criterion described in the literature, which is essential to enable interoperability among nursing vocabularies and to ensure consistent descriptions of nursing phenomena and actions across different settings [3, 6, 17–21].

The terms mapping, cross-mapping, linking, and cross-walk are often used synonymously in the literature [21, 22]. Mapping techniques aim to match or relate the meaning of terms in one terminological system with the concepts of the same meaning in another vocabulary [22]. 

The purpose of this study was to evaluate the inclusiveness and expressiveness of the nursing diagnosis axis of the ATIC terminology by cross-mapping its concepts with the ones in the NANDA-I taxonomy. 

The research questions formulated for this study were the following.

1. To what extent can the ATIC nursing diagnosis be mapped to the NANDA-I nursing diagnosis and vice versa?
2. Are there ATIC nursing diagnoses that cannot be mapped to NANDA taxonomy?
3. Are there NANDA-I nursing diagnoses that cannot be illustrated with the diagnosis axis of the ATIC terminology?

## 2. Methods

### 2.1. Design

The study applied a descriptive design, with a cross-sectional, bidirectional cross-mapping strategy. 

### 2.2. Sample

The objects of this study were the nursing diagnosis concepts from the two controlled vocabularies reported above. ATIC concepts under development or refinement at the time of starting the study were not included.

The sample included 728 nursing diagnosis concepts from the two terminologies: 201 from the NANDA-I taxonomy (2009–2011) and 527 concepts from the diagnosis axis of the ATIC terminology. Both controlled vocabularies were consistent in terms of the proportion of actual and risk nursing diagnosis (Table 1). The term *Actual* (nursing diagnosis) refers to the presence of a problem manifested by different signs, symptoms, or other cues, at the completion of the nursing assessment. The term *Risk* (nursing diagnosis) involves vulnerability because of the presence of risk factors, meaning the patient is likely to develop a problem and preventive interventions are required to avoid it and to minimize vulnerability.

### 2.3. Data Procedures

Criteria were established to systematically address the cross-mapping in accordance with the recommendations found in the literature [18–22], considering that cross-mapping had to be carried out based on the meaning of the concepts, using their definitions. The terms could be the same, but only meanings were considered, independently of the labeling. 

Different mapping categories were predefined for the data collection and were also used as main outcome measures:

- cross-mapping to identify positive connections: one-to-one (1 : 1),
- cross-mapping to identify negative connections: one-to-zero (1 : 0),
- cross-mapping to identify hierarchical connections: many-to-one (*n* : 1) and one-to-many (1 : *n*).

A positive connection (1 : 1) is considered when a concept in a terminology perfectly matches or has equivalence with the meaning of a term in the other vocabulary.

A negative connection (1 : 0) is defined by the presence of a concept in the first vocabulary, missing in the second one.

A hierarchical connection (*n* : 1 or 1 : *n*) implies that different concepts exist in a language system that refer to a concrete concept in the other vocabulary and vice-versa. 

A short standardized data collection sheet was designed to document the mapping category identified for each concept. 

Independent cross-mapping was performed by each researcher, mapping first the NANDA-I Taxonomy to the diagnosis axis of the ATIC terminology and then reversely. The three raters were registered nurses, with a mean of 27 years in nursing practice (range 22 to 37). Two of them held Master's degrees and were associated lecturers at a public university school of nursing. The other had more than 15 years of experience in the field of medical documentation. All had at least five-year experience as superusers of the electronic health record systems in the hospital setting.

The mapping procedure was conducted considering the multiaxial structure of both vocabularies, exploring the meaning of the precoordinated terms but also taking into account the availability of complementary concepts on the different axes (subject, diagnosis status, or location). 

To increase the reliability of the method, cross-mapping was performed twice, first in September 2011 and then in January 2012. The first cross-mapping was considered as a pilot test, and the second was considered for the final analysis. Discrepancies were resolved by consensus.

Permission for the study was obtained from the institution executive board. Permission to use NANDA-I taxonomy (2009–2011) was obtained from the owner of the rights in the country.

Data were processed onto an Excel spreadsheet (Microsoft, Redmond, WA) and revised to identify potential processing errors. Descriptive statistics were used to complete the analysis of the main outcomes. Interrater reliability was calculated considering the percentage of agreement of the raters' results and using Cohen's kappa statistic.

## 3. Results

### 3.1. Agreement of the Raters' Judgements

The percentage of agreement of the raters' mappings reached 97.8%, with no need for further consensus. A discussion session was conducted to solve the 2.2% disagreement, and consensus was established in all cases. Cohen's kappa statistic was calculated as a randomly adjusted agreement measure, resulting in a Kappa value of 0.89. Overall, interrater reliability was very high.

### 3.2. Main Outcome Analysis

The final analysis included the 728 nursing diagnosis concepts from both terminological systems; most of them were actual nursing diagnoses (70% from the ATIC terminology and 72.7% from NANDA-I taxonomy). 

From a total of 201 NANDA-I nursing diagnoses, 121 (60.1%) could be mapped to concepts on the diagnosis axis of the ATIC terminology, 42 (20.8%) obtaining a one-to-one connection. Hierarchical connections accounted for 39.3%, and negative connections were identified in 39.9% of the cases.  Table 2 shows detailed mapping results. 

In the reverse mapping, from the diagnosis axis of the ATIC terminology to the NANDA-I taxonomy, 375 concepts (71.1%) could be illustrated, mainly matching into many-to-one connections (61.2%). Half of the positive and hierarchical connections (54.6%) were possible only when adding a complementary concept from another axis of the NANDA-I taxonomy, mainly the “Subject” and “Location” axes. Negative connections accounted for 28.9% because these concepts were missing in the NANDA-I taxonomy (Table 2). 

Sample nursing diagnoses falling within each connection category are presented in Table 3.

## 4. Discussion

This study aimed to examine the inclusiveness and expressiveness of the concepts within the diagnosis axis of the ATIC terminology by cross-mapping them with the NANDA-I taxonomy. The results show that more than 60% of concepts were bidirectionally connected.

With regard to the evaluation of the criterion validity, these results show that, to a moderate degree, the diagnosis axis of ATIC includes terms of the same meaning for the description of the nursing diagnosis as those in the NANDA-I taxonomy. However, some issues should be taken into account.

First, the results of this study should be discussed bearing in mind the basic difficulties involved in the mapping procedures, as described in the literature [22]. 

Second, in the 2009–2011 version of the NANDA-I taxonomy used for this study, the diagnoses included are mainly pre-coordinated concepts [23]. Atomic level cross-mapping of the concepts would probably have shown better results, but at present, the “focus” axis of the NANDA-I taxonomy includes terms (units of language) at atomic level, but not concepts (meanings or units of thought), or they are not available. 

Atomic or kernel concepts are fundamental concepts aimed to facilitate the generation of compositional expressions in a controlled vocabulary [1, 13, 24, 25]. As Whittenburgh states: “*Data at the atomic level represents a basic data form, essentially the smallest meaningful unit in any system*” [26]. 

The mapping procedure used in our study was based on the assumption that the definition of a pre-coordinated diagnosis in the NANDA-I taxonomy would reflect the meaning of the concept in its focus axis. 

Third, an effect was observed in relation to the granularity of the concepts. Granularity is “*the level of detail that a term in a standardized terminology represents*” [27].

Controlled terminologies may include concepts with different levels of granularity, from very abstract concepts to very specific ones. For direct patient care the lowest level of abstraction is recommended [27]. The observed effect was that there were many general concepts, for example, *Anxiety*, that may implicitly contain other more specific diagnoses such as *Separation anxiety*.

Although research in the field of diagnostic expertise in nursing is in the early stages, differences between novice and proficient nurses' ability to make accurate judgments concerning the state of the patient are expected to be found, so different degrees of abstraction are probably needed within any terminology to properly cover diverse levels of nursing clinical expertise [28, 29].

Fourth, hierarchical many-to-one connections (*n* : 1) found in the reverse mapping from the ATIC terminology to the NANDA-I taxonomy may indicate that the diagnostic concepts in this taxonomy are slightly too abstract to properly detail some of the nursing judgments on patients' responses in the practice setting. Similarly, the low number of many-to-one connections from the NANDA-I to this new interface terminology may be an indicator of the specificity of the ATIC concepts needed for direct patient care. 

Fifth, “Readiness for enhanced (…)” diagnoses in the NANDA-I taxonomy are not considered diagnostic concepts in the ATIC terminology because they are conceptualized as outcomes cues. This might explain a 33% of negative connections (1 : 0) from NANDA-I to the ATIC terminology.

In a previous study, which cross-mapped the NANDA-I taxonomy with the Omaha system and the Home Health Care Classification, the researchers found that only 15.9% of concepts achieved a one-to-one match (1 : 1) and that 61.1% were hierarchically related [30]. Focusing on the total number of nursing diagnoses explored, the results of our study are not so different.

The present study has some limitations. The first are those inherent to the descriptive cross-mapping design, which prevents extended statistical analysis. The others are as follows. 

The correct mapping of concepts requires a 1 : 1 connection between the terms of two vocabularies. A positive connection (1 : 1) is the ideal relationship in cross-mapping but, as noted in the literature, it is a rare event [22].

Methodological studies on cross-mapping were not found in the literature. The mapping procedure in this study was based on previous assays in which different nursing vocabularies were mapped; this fact may have introduced a bias in the results. 

Pilot testing of the data collection sheet and the mapping procedure was performed but no additional method was used to verify the quality of the mapping; this should also be considered a limitation.

Further research is needed to demonstrate the inclusiveness and expressiveness of the diagnosis axis of the ATIC terminology. As suggested elsewhere, mapping with other nursing terminological systems—especially with ontology-based reference terminologies like the International Classification of Nursing Practice (ICNP), which is designed considering the power of the atomic level data—is probably needed to demonstrate that the diagnosis axis of the ATIC terminology consistently meets the validity criterion of cross-mapping [31]. 

Finally, to date the research in the field of nursing controlled vocabularies has demonstrated that “*not all terminologies serve all purposes equally well*” [2], so it is probably time to realize that some questions need to be responded such as why nursing classifications and taxonomies are being used as interface terminologies in healthcare computer-based systems.

## 5. Conclusions

Valid, reliable, comprehensive, easy-to-use, nursing interface terminologies are needed in nursing practice. Mapping interface terminologies to other controlled vocabularies enhances interoperability, facilitates health information exchange, and ensures consistent descriptions of nursing care across different specialties, settings, and countries.

The results of this study suggest that the diagnosis axis of this nursing interface terminology meets the validity criterion of cross-mapping when carried out from and to the NANDA-I taxonomy. 

## Figures and Tables

**Table 1:** Distribution of the nursing diagnosis.

	NANDA-I taxonomy		ATIC terminology	
	*N*	%	*N*	%
Risk diagnosis	55	27.3	158	30.0
Actual diagnosis	146	72.7	369	70.0

**Table 2:** Main cross-mapping results.

	Mapping from NANDA-I to ATIC		Mapping from ATIC to NANDA-I	
Connections	*N*	%	*N*	%
One-to-one (1 : 1)	42	20.8	42	8.0
One-to-zero (1 : 0)	80	39.9	152	28.9
One-to-many (1 : *n*)	64	31.8	10	1.9
Many-to-one (*n* : 1)	15	7.5	323	61.2

**Table 3:** Sample nursing diagnoses.

	NANDA-I nursing diagnosis	ATIC nursing diagnosis axis
One-to-one (1 : 1)	Neonatal jaundice	Neonatal jaundice
	Diarrhea	Diarrhea
	Fatigue	Fatigue

One-to-zero (1 : 0)	Readiness for enhanced nutrition	—
	Energy field disturbance	—

One-to-many (1 : *n*)	Anxiety	Anxiety
		Separation anxiety

Many-to-one (*n* : 1)	Functional urinary incontinence	Urinary Incontinence
	Urge urinary incontinence	
